# Long-Term Outcomes and Predictors of Childhood-Onset Schizophrenia: A Naturalistic Study of 6-year Follow-Up in China

**DOI:** 10.3389/fpsyt.2021.679807

**Published:** 2021-07-30

**Authors:** Zheng Liangrong, Zhang Guican, Zhu Qi, Yang Weirui, Zhang Yaqi, Li Tong, Liang Wenjing, Zhang Ming, Guan Nianhong

**Affiliations:** Department of Psychiatry, The Third Affiliated Hospital of Sun Yat-sen University, Guangzhou, China

**Keywords:** schizophrenia, childhood-onset, outcome, follow-up, predictors

## Abstract

**Objectives:** The long-term outcome of childhood-onset schizophrenia (COS) and its influencing factors remain unclear. The current study aimed to assess the long-term outcomes of COS and identify possible outcome predictors.

**Methods:** We retrospectively investigated 276 patients with COS. Diagnosis made according to the ICD-10 criteria for schizophrenia, and the age of the first onset was ≤ 14 years. Follow-up was completed for 170 patients, with a median follow-up period of 5.6 years. Outcome variables included occupational/education status and readmission. Spearman correlation was performed to assess the relationship between predictors and outcome variables. Binary logistic regression was conducted to detect possible predictor variables for outcome variables.

**Results:** At the end of the follow-up, 89 patients (52.3%) were at school, 70 patients (41.2%) were employed, and only 11 patients (6.5%) were dropped out of school or unemployed. The duration to the first admission and depressive symptoms were identified as predictors of occupational/educational status. The length of follow-up and obsessive-compulsive symptoms (OCS) were distinguished as predictors of readmission. Duration to the first admission and length of follow-up were risk factors, and depressive symptoms and OCS were protective factors for the outcomes of COS.

**Conclusion:** We found a favorable long-term outcome on occupational/education status in COS, and depressive symptoms and OCS may be associated with more positive long-term outcomes in COS. Our findings suggest that COS patients may benefit from early intervention and require appropriate treatment.

## Introduction

Childhood-onset schizophrenia (COS) is a severe chronic mental illness (1). It can be reliably diagnosed using the same diagnostic criteria as adult-onset schizophrenia (2). The definition of COS varies between studies, with an age of diagnosis being considered before 12- and 15-years-old (3). The incidence of COS is <0.04% (4). As COS is a very rare disease, little is known about it. Clinicians need to understand the outcome of COS and its predictors better to recognize and treat it better.

In most of the studies on COS outcome, the sample size is small, the length of follow-up is different, and the outcome is inconsistent with varying measures of outcome or remission criteria. In some previous studies, favorable outcomes of COS ranged from 15.8 to 56% (5–7). Occupational/educational status and readmission are two of the measures that had been used to evaluate the outcome of COS (8). A follow-up study (7) found that COS patients had poor occupational or education function, with 73.7% of the sample never graduating from school and 71.1% unemployed. Another study of 18 children with schizophrenia or schizotypal personality disorder found 38.8% of the sample readmission (8). However, to our knowledge, the long-term outcome base on occupational/educational status or readmission of COS diagnosed according to the International Classification of Diseases, 10th Revision (ICD-10) remains unclear in China.

There are few studies on the outcome predictors of COS. Previous studies have found some outcome predictors of early-onset schizophrenia (EOS), which may provide a reference for outcome predictors of COS. EOS is defined as schizophrenia onset before 18 years old (9) and includes COS. Some factors affecting the outcome of EOS have been identified. These factors include gender, IQ, type of onset, premorbid adjustment, medication compliance, positive symptoms, negative symptoms, follow-up periods, disease course, duration of untreated psychosis (DUP), family history of schizophrenia, and family history of psychosis (3, 9–13). Predictors of outcomes differed according to outcome criteria (8). It is unknown whether these factors influence the outcomes of COS based on occupational/educational status or readmission.

Comorbid symptoms such as depressive, excitement, anxiety, and obsessive-compulsive symptoms (OCS) are common in schizophrenia (14–17) and be associated with the outcome of schizophrenia (18–21). Antipsychotic polypharmacy is associated with more unsatisfactory outcomes (22). However, it remains unclear whether comorbid symptoms and antipsychotic polypharmacy are predictors of COS outcomes.

This study aimed to assess the long-term outcomes based on occupational/educational status or readmission and their possible influencing factors of COS diagnosed according to the ICD-10 in a cohort of patients in China.

## Methods

**Figure 1:**
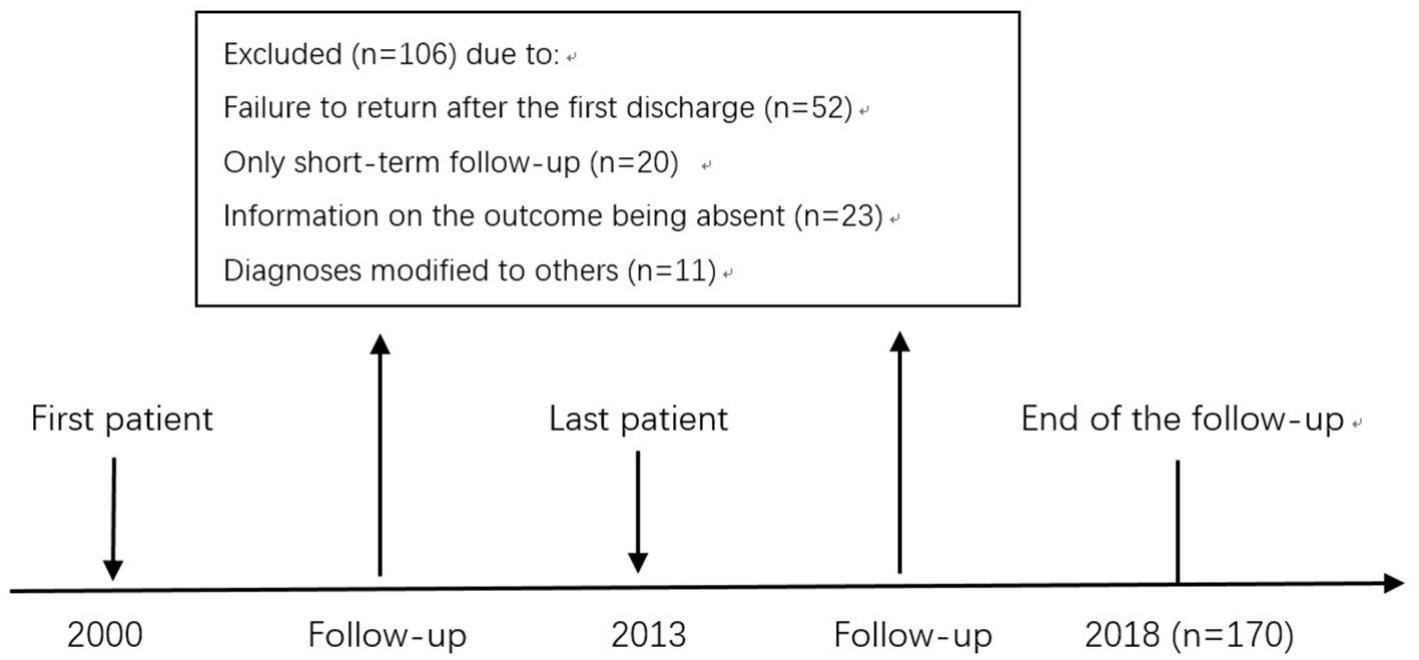
Timetable and design of follow-up study of COS^a^. ^a^The inclusion criteria for this study include the age of first onset ≤ 14 years old, fulfilling the criteria for schizophrenia according to the ICD-10, no comorbidities, no primary organic mental disorder, no drug and/or alcohol-induced psychosis, and no intellectual disability.

This retrospective cohort study was carried out from January 2018 and March 2019 at the Third Affiliated Hospital of Sun Yat-sen University, Guangzhou, Guangdong Province. The timetable and design of the study are shown in Figure 1.

### Participants

The sample included 276 patients, age of first onset ≤ 14 years old, fulfilling the criteria for schizophrenia according to the ICD-10, and admitted to the Department of Psychiatry at the Third Affiliated Hospital of Sun Yat-sen University for the first time, between January 2000 and December 2013.

After admission, patients had been conducted an accurate history and a physical and thorough neurologic examination, relevant laboratory and imaging tests to rule out mental disorders caused by organic diseases. The related laboratory and imaging tests included blood count, urinalysis, liver, kidney function tests, serum chemistries, electrolytes, clotting studies, thyroid function test, chest radiograph, electrocardiogram, electroencephalography, computed tomography scan or magnetic resonance imaging of the brain, etc. After a thorough evaluation by a chief physician/an associate chief physician, and an attending physician, the patient was diagnosed with childhood-onset schizophrenia according to the ICD-10. The admission diagnosis was sustained at discharge. There were no comorbidities. Primary organic mental disorder, drug and/or alcohol-induced psychosis, intellectual disability were excluded.

Regular follow-up had been completed for 170 patients, with 106 patients being excluded due to one of the following reasons: a failure to return after discharge, only short-term follow-up being possible, information on the outcome being absent during the follow-up period, or the diagnoses had been modified to others (Figure 1). Comparing the drop-out group to those who had completed the follow-up, gender, age of the first onset, family history of psychiatric illness, show no significant difference (Table 1). Patients were followed up at least once every 2–3 months after first discharge during the follow-up period. None of the subjects who completed the follow-up recovered from the initial COS diagnosis and all were still diagnosed with schizophrenia.

**Table 1:** Comparisons of study sample and those lost to recruitment^*a*^.

	**Gender^**b**^**	**Age of the first onset (years)**	**Family history of psychiatric illness^**c**^**
Mann–Whitney *U*	8,659	8,194.5	8,332.5
Wilcoxon W	23,194	13,865.5	22,867.5
*Z*	−0.63	−1.302	−1.333
Asymp. Sig. (2-tailed)	0.529	0.193	0.182

a *The nonparametric Mann–Whitney U-test was used for comparison of the study sample and those lost to recruitment*.

b *Value of Gender. Male = 1, Female = 2*.

c *Value of family history of psychiatric illness. No family history of psychiatric illness = 0, Family history of schizophrenia = 1, Family history of other psychiatric illness = 2*.

The primary treatment for patients was pharmacotherapy. Other therapies included psychotherapeutic and early psychosocial intervention, such as education and support of the family about this disorder. Antipsychotics were used for all patients. Some patients also receive mood stabilizers, antidepressants, or anxiolytics from the first admission to the end of the follow-up (Table 2).

**Table 2:** Demographics and clinical characteristics of follow-up sample^a^.

	**Follow-up sample (** ***n*** **=** **170)**				
	***N***	**%**	**Range**	**Median**	**Inter quartile range**
**Gender**					
Male	82	48.2			
Female	88	51.8			
**Family history of psychosis**					
No family history of psychosis	127	74.7			
Family history of schizophrenia	10	5.9			
Family history of other psychosis	33	19.4			
Age at first admission (years)			5–14	14	13–14
Age at first onset (years)			5–14	13	12–13
Age at the end of the follow-up (years)			7–30	18	15–21
Duration to the first admission^b^ (months)			1–120	6	2–13
Duration of the first admission (days)			1–212	36	26–51
Length of follow-up (years)			0.2–16.8	5.6	3.0–7.7
DUP at first onset (months)			0.1–120	4	1–12
**Hallucinations** ^**c**^	120	70.6			
Auditory hallucination	117	68.8			
Visual hallucination	18	10.6			
Olfactory hallucination	4	2.4			
Tactile hallucination	1	0.6			
Visceral hallucination	1	0.6			
**Delusions** ^**c**^	117	68.8			
Delusion of persecution	102	60.0			
Delusion of reference	67	39.4			
Experience of being revealed	39	22.9			
Feeling of being controlled	19	11.2			
Delusion of being loved	2	1.2			
Delusion of nonconsanguinity	4	2.4			
**Negative symptoms** ^**c**^					
Affective flattening	96	56.5			
Avolition	91	53.5			
poverty of thought	83	48.8			
**Comorbid symptoms** ^**d**^					
Depressive symptoms followed by Excitement	30	17.6			
Excitement followed by Depressive symptoms	7	4.1			
Depressive symptoms	17	10.0			
Excitement	19	11.2			
Irritability	10	5.9			
OCS	23	13.5			
Anxiety symptoms	2	1.2			
**Antipsychotic therapy** ^**e**^					
Use 1 antipsychotic	98	57.6			
Use 2 antipsychotics	69	40.6			
Use 3 antipsychotics	3	1.8			
**Other medications** ^**f**^					
Use mood stabilizers	55	32.4			
Use antidepressants	35	20.6			
Use anxiolytics	6	3.5			

a *The inclusion criteria of the follow-up sample include the age of first onset ≤ 14 years old, fulfilling the criteria for schizophrenia according to the ICD-10, no comorbidities, no primary organic mental disorder, no drug and/or alcohol-induced psychosis, and no intellectual disability*.

b *Duration to the first admission was defined as the duration from the onset of characteristic symptoms (meeting symptoms criteria of schizophrenia according to the ICD-10) to the first day of the first admission*.

c *Hallucinations, delusions, and negative symptoms were collected from the first admission data*.

d *Comorbid symptoms were collected from the first admission to the end of the follow-up*.

e *Antipsychotic therapy was collected from the first admission data*.

f *Other medications were collected from the first admission to the end of the follow-up*.

Retrospective diagnostic evaluation of the patients was carried out by two experienced clinicians based on the patients' medical records during the follow-up period. Eleven patients were excluded from the follow-up sample due to the change in diagnosis (Eight patients were diagnosed with schizoaffective disorder, two as bipolar disorder, and one as obsessive-compulsive disorder). In contrast, the other patients of the follow-up sample had no change in diagnosis.

### Assessment

All first admission data, including socio-demographic data, duration to the first admission, first onset symptoms, treatment history, and family history, were extracted from inpatient medical records.

Information on the patient was obtained from inpatient medical records and outpatient medical records. Face-to-face follow-up meeting records were used to get the following information: duration of untreated psychosis (DUP) at the first onset, comorbid symptoms from the first admission to follow-up, inpatient duration, education/occupational status. Comorbid symptoms include emotional symptoms (depressive symptoms, excitement, depressive symptoms followed by excitement, excitement followed by depressive symptoms, or irritability), OCS, and anxiety symptoms.

From the scripts of the medical records (including inpatient medical records, outpatient medical records, and face-to-face follow-up meeting records), we extracted the information relevant to the symptom(s) from first-onset to the end of the follow-up, including the specific features of the symptom and occurrence time (e.g., OCS). The symptom descriptors reflected the terms used in the medical records. For instance, from the medical record, “The patient said that 1 month ago he began to check the door lock and switch at home repeatedly. He was aware that the above behavior is not necessary. But he could not control, and felt painful.” The information related to the symptoms was extracted as follows:

Symptom = Compulsive checking.

Occurrence time = 1 month.

Specific feature(s) = OCS.

DUP was defined as the time interval from the first sign of abnormality in the patient to the beginning of antipsychotic treatment, determined from information in inpatient and outpatient medical records. Duration to the first admission was defined as the duration from the onset of characteristic symptoms (meeting symptoms criteria of schizophrenia according to the ICD-10) to the first day of the first admission.

Two specialists had been arranged to check the data extraction to ensure its reliability during the study period.

### Outcome Measures

Outcomes were evaluated in the follow-up sample. The outcome measures include occupational/education status and readmission. Occupational/educational status is defined and quantified as at school or employed, dropped out of school, or unemployed. Readmission is defined and quantified as a yes/no measure of readmission. In our study, readmission was all for actual relapse of psychosis.

### Statistical Analysis

Patient demographics, clinical characteristics, and outcomes were reported mainly in a descriptive way. Statistical analyses were performed using SPSS version 22.0. All tests were two-tailed, with *p* < 0.05 being considered statistically significant.

The Kolmogorov–Smirnova test showed that continuous variables, including age at the first admission, age at the first onset, age at the end of the follow-up, the duration to the first admission, duration of the first admission, length of follow-up, DUP at first onset did not conform to the normal distribution (*P* < 0.05). Therefore, continuous data are expressed as the median, interquartile range.

As gender and family history of psychosis are categorical data, and the age of the first onset did not conform to the normal distribution. Non-parametric Mann–Whitney *U*-test was used to examine group differences in these variables.

The occupational/education status and readmission were considered the outcome variables in the study. We examined some factors as potential predictors of outcomes. These factors include the family history of psychosis, DUP at the first onset, age of onset, age at first admission, age at the end of follow-up, duration to the first admission, duration of the first admission, length of follow-up, main symptom(s) at the first admission, medicinal therapy, emotional symptoms, OCS, and anxiety symptoms. The value for these variables is shown in Table 3. Spearman correlation was carried out to examine the relationship between the predictor variables and the outcome variables (occupational/education status and readmission). Binary logistic regression with “enter” method was used to detect possible predictor variables for outcome variables (occupational/education status and readmission).

**Table 3:** Possible predictors of COS outcomes and their value.

**Possible predictors**	**Variable**	**Value**
Occupational/education status	Y1	No = 0, Yes = 1
Readmission	Y2	No = 0, Yes = 1
Gender	X1	Male = 1, Female = 2
Age of the first onset	X2	Original data
Age of the first admission	X3	Original data
Age at the end of follow-up	X4	Original data
DUP	X5	Original data
Duration of first admission	X6	Original data
Duration to the first admission	X7	Original data
Depressive symptoms followed by excitement	X8	No = 0, Yes = 1
Excitement followed by depressive symptoms	X9	No = 0, Yes = 1
Depressive symptoms	X10	No = 0, Yes = 1
Excitement	X11	No = 0, Yes = 1
Irritability	X12	No = 0, Yes = 1
OCS	X13	No = 0, Yes = 1
Anxiety symptoms	X14	No = 0, Yes = 1
Auditory hallucination	X15	No = 0, Yes = 1
Visual hallucination	X16	No = 0, Yes = 1
Other hallucination	X17	No = 0, Yes = 1
Delusion of persecution	X18	No = 0, Yes = 1
Delusion of reference	X19	No = 0, Yes = 1
Experience of being revealed	X20	No = 0, Yes = 1
Feeling of being controlled	X21	No = 0, Yes = 1
Other delusion	X22	No = 0, Yes = 1
Antipsychotic therapy	X23	1 = Antipsychotic monotherapy, 2 = Antipsychotic polypharmacy
Family history of psychosis	X24	No family history of psychosis = 0, Family history of schizophrenia = 1, Family history of other psychosis = 2 (defined as nominal variables, dummy variables were generated automatically by software when included in the model)

## Results

### Demographics and Clinical Characteristics of the Follow-Up Sample

We followed up with 170 COS patients regularly for further assessment. Demographics and some clinical characteristics of follow-up patients are shown in Table 2.

### Main Symptom(s), Comorbid Symptoms, and Medicinal Therapy of the Follow-Up Sample

Main symptom(s), comorbid symptoms, antipsychotic therapy at first admission, and other medications from the first admission to the final follow-up in the follow-up sample are shown in Table 2. The most common being auditory hallucinations. The most common being delusions of persecution. Comorbid symptoms included emotional symptoms, OCS, and anxiety symptoms from the first admission to the end of the follow-up in the follow-up sample, as shown in Table 2.

### Outcomes of COS

The outcomes of COS at the follow-up stage are shown in Table 4. Most patients (93.5%) were at school or employed. Among the 89 patients at school, four were studying for a master's degree, and two were studying in the United States. There are 44 patients readmitted, accounting for 25.9% of the follow-up sample.

**Table 4:** Outcomes of COS.

	**Follow-up sample (** ***n*** **=** **170)**	
	***N***	**%**
**Occupational/education status**		
At school or employed	159	93.5
At school	89	52.3
Employed	70	41.2
Dropped-out of school or unemployed	11	6.5
**Total number of hospital admission**		
1	126	74.1
2	34	20.0
3	7	4.1
4	2	1.2
5	1	0.6
**Readmission**		
Yes	44	25.9
No	126	74.1

### Outcome Predictors of COS

Spearman correlation of occupational/education status and other factors, and Spearman correlation of readmission and other factors are shown in Table 5.

**Table 5:** Spearman correlation of COS outcomes and other factors.

**Items**	**Variables**	**Spearman *r***	***P***
**Occupational/education status**	Duration to the first admission	−0.204	0.008
	Depressive symptoms	−0.151	0.049
	OCS	−0.176	0.022
	Other hallucinations^a^	−0.209	0.006
**Readmission and other factors**	Length of follow-up (months)	0.185	0.015
	DUP	−0.154	0.045
	OCS	0.198	0.010

a *Other hallucinations included olfactory hallucination, tactile hallucinations, and visceral hallucinations*.

Binary logistic regression with “enter” method was performed, using occupational/education status as the dependent variable. Independent variables included in the model were: course of the disease, depressive symptoms, OCS, and other hallucinations. The duration to the first admission and depressive symptoms were found to be significantly associated (*P* < 0.05) with occupational/education status (Table 6). Duration to the first admission was a risk factor, and depressive symptoms were a protective factor for occupational/education status.

**Table 6:** Binary logistic regression analyses of COS outcomes.

**Items**	**Variables**	**B**	**S.E**	**Wals**	**Exp (B)**	**95%C.I. of Exp (B)**	***P***
**Occupational/education status**	Duration to the first admission	−0.035	0.014	6.400	0.966	0.940–0.992	0.011
	Depressive symptoms	−2.015	0.825	5.968	0.133	0.026–0.671	0.015
	OCS	−1.353	0.780	3.006	0.258	0.056–1.193	0.083
	Other Hallucinations^a^	−1.942	1.066	3.318	0.143	0.018–1.159	0.069
	Constant	4.112	0.638	41.610	61.083		0.000
**Readmission**	Length of follow-up	0.010	0.004	6.970	1.011	1.003–1.018	0.008
	DUP	−0.021	0.016	1.670	0.979	0.949–1.011	0.196
	OCS	1.203	0.481	6.242	3.330	1.296–8.555	0.012
	Constant	−1.856	0.383	23.475	0.156		0.000

a *Other hallucinations included olfactory hallucination, tactile hallucinations, and visceral hallucinations*.

Another binary logistic regression with “enter” method was performed, using readmission as the dependent variable. Independent variables included in the model were: length of follow-up, DUP at the first onset, and OCS. Length of follow-up and OCS were found to be significantly associated (*P* < 0.05) with readmission (Table 6). Length of follow-up was a risk factor, and OCS was a protective factor for occupational/education status.

## Discussion

### General Outcome

Our study has found that COS has a more favorable outcome after a long-term follow-up than some previous studies, particularly regarding occupational/ education status. Remschmidt et al. (7) investigated the outcome of COS patients after a mean follow-up of 42 years. They found that 7.9% of patients graduated from secondary school, 18.4% graduated from elementary school, 73.7% failed to graduate from any school, 5.2% were employed, 23.7% were receiving a pension, and 71.1% were unemployed.

Compared to studies of EOS outcomes involving children and adolescents with schizophrenia, our results are also more optimistic. One retrospective study (23) showed that at a mean follow-up of 13.4 years, only 18.5% of EOS patients were employed, and 18.5% of patients had not graduated from school. Lay et al. (24) found that after a mean follow-up of 10 years, 57% of the EOS patients had at least moderate impairment of vocational skills and could not achieve their premorbid educational or occupational goals.

There may be some possible reasons for the better occupational/education functioning of COS patients in our study. First, Chinese culture attaches great importance to family relationships. Family members support and encourage each other, manage their problems, and reduce the burden together. And COS patients whose families cooperate well had been found to have better outcomes (9). Second, COS patients admitted to our department are strongly encouraged to achieve their educational or occupational goals. However, it is worth noting that the drop-out rate in our study was high. A high drop-out rate was associated with more poor outcomes (3). Therefore, our study may underestimate the percentage of poor prognosis, and the outcomes of our sample may be more positive.

Readmission was used as another measure of outcome in our study. Previous studies have paid little attention to the readmission of COS. Therefore, we compared the results of studies on readmission of EOS, which includes the COS group. Vyas and Hadjulis et al. (13), investigating the outcome of EOS patients after a mean follow-up of 4 years, found that the subjects had an average of 2.09 ± 1.44 hospital admissions during the follow-up period. A 3-year follow-up study of EOS (25) in China found that 58.4% of the follow-up sample were hospitalized twice or more. Another study (26) showed that after a mean follow-up of 8 years, 71% of EOS spectrum psychosis patients had been rehospitalized. We found that the COS sample had an average of 1.34 ± 0.67 hospital admissions, and only 25.9% had been readmitted during the follow-up period. Our results are more favorable than the results of these previous studies of the EOS group.

In summary, compared to most previous studies, we found that COS patients have a more favorable long-term outcome. There may be some reasons for the better prognosis of COS in our research. The treatment of schizophrenia has improved due to the widespread use of second-generation antipsychotics, the development of community services, and the deinstitutionalization movement over recent decades in China (25). The patients in our study had received medication, psychotherapy, and psychosocial intervention. These treatments contribute to better outcomes for COS patients (27).

### Possible Predictors for the Occupational or Education Status in COS

We found that depressive symptoms and the duration to the first admission were protective factors for a positive occupational/education status in COS.

Depressive symptoms are common in first-episode psychosis (28) and present up to 70% of patients with schizophrenia (29). We found that the prevalence of depressive symptoms in COS was 10%. This prevalence is much lower than the model rate of 25% depression in schizophrenia (30) and 30.8% in EOS (23). However, in our study, these depressive symptoms did not include depressive symptoms accompanied by excitement symptoms.

The effect of depressive symptoms on schizophrenia varies greatly and depends on the stage of the disease they occur (31). Depressive symptoms in schizophrenia's acute phase have been associated with a more favorable outcome and good treatment response, while depressive symptoms that persist or emerge after the acute phase appear to be predictive of a more unsatisfactory outcome (28, 32).

In our study, depressive symptoms were reported in COS patients in the acute and chronic phases of the disease, with the majority in the acute phase. Depressive symptoms in the acute phase of schizophrenia may predict more significant improvements in positive and negative symptoms (32). Positive symptoms and negative symptoms can lead to poor social and occupational functioning (24, 32, 33). These might explain why depressive symptoms were associated with better occupational or educational status in our study.

We found that a longer duration to the first admission was a risk factor for poor occupational or education status in COS. COS development is generally more insidious than EOS, making it difficult to identify early (34). Besides, insidious onset and long duration of the first episode have been noted as predictors for a chronic long-term course of childhood or adolescence onset schizophrenia (9). So it is essential to identify COS and initiate treatment early to improve outcomes of COS.

### Possible Predictors for Readmission of COS

The prevalence of OCS has ranged from 10 to 64% (35) and a mean of 30.7% (14) in schizophrenia patients. We found that the prevalence of OCS was 13.5% in COS. This prevalence is much lower than the 26% of adolescent schizophrenia (35) and a mean of 30.7% in schizophrenia patients (14).

OCS can be seen in many phases of schizophrenia, starting from the at-risk mental state to the chronic, stabilization, and deficit phase (21). OCS can be present in adolescents, adults, and elderly patients with schizophrenia (36, 37). We found OCS in COS as well.

Studies regarding the impact of OCS on schizophrenia are conflicted. Some studies have reported more severe global, positive, and negative psychotic symptoms, as well as depressive symptoms, social dysfunction, lower quality of life, and worse premorbid functioning in schizophrenia due to OCS (38–40). Other studies, however, have reported a higher rate of occupation, lower prevalence of comorbid psychiatric disorders, less frequent hospitalization, longer duration of education, less formal thought disorder, a less severe flat affect, better cognitive function, and better global functioning (41–43).

The underlying causes of conflicting results may be related to the phase-dependent effects of OCS on functioning. For instance, OCS may have protective effects in the early stages of schizophrenia and deleterious effects in the later stages (44). Besides, the severity of obsessive-compulsive features may affect social functioning in schizophrenia, and mild OCS may protect against worse social functioning; however, OCD may worsen social functioning (40).

We found that OCS was a protective factor for hospital readmission in COS. It is consistent with a previous study showing that hospitalization frequency in patients with schizophrenia with OCS was lower than those without OCS (43). Our study did not distinguish the onset phase of OCS and the severity of OCS. We supposed that there might be a predominance of OCS in the early stage and less severe cases in our study, and these lead to the finding that OCS is a protective factor for readmission.

We found that a longer follow-up duration is a risk factor for readmission in COS. Remberk et al. (45) reported that a longer length of follow-up was associated with a higher probability of clozapine therapy and a lack of occupational activity, suggesting worse outcomes in early-onset schizophrenia spectrum psychoses. Moreover, a systematic review (3) of EOS indicated that a longer follow-up period was associated with poor outcome. Furthermore, schizophrenia has been found to cause a progressive decrease in white matter and gray matter, which correlates with cognitive impairment (46). The previous findings suggest an unfavorable course of the disease, and the risk of readmission may increase with the increase of follow-up duration.

### Family History of Psychosis in COS

Few studies have reported the proportion of family history of psychosis in COS. Green et al. (47) had reported that 26.3% of COS patients had one or both parents hospitalized for mental illness. In their study, other relatives (such as siblings or unhospitalized patients) with a positive family history were not included when evaluating the family history of mental illness. The proportion of family history of psychosis in our study was nearly a quarter which was low compared to Green's study. In China, perceived stigma may prevent disclosing cases of mental illness within the family even when these are known. In addition, mental illness may not be detected without a direct clinical interview. So it is reasonable to assume that the actual proportion of family history of psychosis in our study would be higher.

### Strengths and Limitations

To our knowledge, this is the first Chinese long-term follow-up study of a large sample of COS patients using the ICD classification system, making our results more comparable to research in western countries. Furthermore, we have paid attention to the effect of clinical symptoms, including comorbid symptoms such as depressive symptoms and OCS, on outcomes in COS. We hope our results will enable clinicians to understand better the impact of different factors on the course of COS, control the symptoms better, and improve patient outcomes.

However, several limitations can be identified in our study. First, this was a cross-sectional study rather than a longitudinal study, resulting in a recall bias of some details and can only reflect the situation at the time of follow-up. Second, the drop-out rate of our study was relatively high. In our study, 52 patients did not return after the first discharge. Previous follow-up studies have reported mortality due to suicide of 15.8% for COS and about 10% for adult schizophrenia (7, 48). Given the prevalence of death by suicide in individuals with schizophrenia, it cannot be ruled out that some patients of our sample may have committed suicide and never return. Therefore, our follow-up samples may not represent the most severely affected individuals of COS. Finally, our sample was recruited from a psychiatric department of a general hospital. As a result of this, compared to a typical mental health hospital, the admission duration of patients in our study is relatively short. Therefore, our sample may not be representative of the whole COS patient group.

## Conclusion

The present study indicates a much more favorable long-term outcome in COS than most previous studies, raising questions around the assumption of a typically poor outcome in COS patients. The results suggest that more attention should be paid to comorbid symptoms such as depressive symptoms and OCS, which leads to a more favorable long-term outcome in COS. The findings also suggest that COS patients may benefit hugely from early intervention and also require adaptive treatment. Our results may destigmatize the COS group and promote a more recovery-oriented therapy approach in this patient group.

## Data Availability Statement

The raw data supporting the conclusions of this article will be made available by the authors, without undue reservation.

## Ethics Statement

The studies involving human participants were reviewed and approved by Ethics Committee of the Third Affiliated Hospital of Sun Yat-sen University. Written informed consent to participate in this study was provided by the participants' legal guardian/next of kin.

## Author Contributions

ZL collected data, analyzed and interpreted data, and drafted the article. GN and ZM made substantial contributions to the conception and design, and finally approved the version to be published. LW, LT, ZY, YW, and ZQ collected data, analyzed, and interpreted data. ZG collected data, revised the article critically for important intellectual content. All authors contributed to the article and approved the submitted version.

## Conflict of Interest

The authors declare that the research was conducted in the absence of any commercial or financial relationships that could be construed as a potential conflict of interest.

## Publisher's Note

All claims expressed in this article are solely those of the authors and do not necessarily represent those of their affiliated organizations, or those of the publisher, the editors and the reviewers. Any product that may be evaluated in this article, or claim that may be made by its manufacturer, is not guaranteed or endorsed by the publisher.
